# Larvicidal, antimicrobial and brine shrimp activities of extracts from *Cissampelos mucronata *and *Tephrosia villosa *from coast region, Tanzania

**DOI:** 10.1186/1472-6882-11-33

**Published:** 2011-04-23

**Authors:** Ramadhani S O Nondo, Zakaria H Mbwambo, Abdul W Kidukuli, Ester M Innocent, Matobola J Mihale, Paul Erasto, Mainen J Moshi

**Affiliations:** 1 Department of Biological and Pre-Clinical Studies, Institute of Traditional Medicine, Muhimbili University of Health and Allied Sciences (MUHAS), P.O. Box 65001, Dar es Salaam, Tanzania; 2 Department of Natural Products Development and Formulation, Institute of Traditional Medicine, MUHAS, P.O.BOX 65001, Dar es Salaam, Tanzania; 3 Department of Physical Sciences, Open University of Tanzania, P.O. Box 31608, Dar es Salaam, Tanzania

## Abstract

**Background:**

The leaves and roots of *Cissampelos mucronata *A. Rich (Menispermaceae) are widely used in the tropics and subtropics to manage various ailments such as gastro-intestinal complaints, menstrual problems, venereal diseases and malaria. In the Coast region, Tanzania, roots are used to treat wounds due to extraction of jigger. Leaves of *Tephrosia villosa *(L) Pers (Leguminosae) are reported to be used in the treatment of diabetes mellitus in India. In this study, extracts from the roots and aerial parts of *C. mucronata *and extracts from leaves, fruits, twigs and roots of *T. villosa *were evaluated for larvicidal activity, brine shrimps toxicity and antimicrobial activity.

**Methods:**

Powdered materials from *C. mucronata *were extracted sequentially by dichloromethane followed by ethanol while materials from *T.villosa *were extracted by ethanol only. The extracts obtained were evaluated for larvicidal activity using *Culex quinquefasciatus *Say larvae, cytotoxicity using brine shrimp larvae and antimicrobial activity using bacteria and fungi.

**Results:**

Extracts from aerial parts of *C. Mucronata *exhibited antibacterial activity against *Staphylococcus aureus*, *Escherichia coli*, *Pseudomonas aeruginosa*, *Salmonella typhi*, *Vibrio cholera*, *Bacillus anthracis*, *Streptococcus faecalis *and antifungal activity against *Candida albicans *and *Cryptococcus neoformans*. They exhibited very low toxicity to brine shrimps and had no larvicidal activity. The root extracts exhibited good larvicidal activity but weak antimicrobial activity. The root dichloromethane extracts from *C. mucronata *was found to be more toxic with an LC_50 _value of 59.608 μg/mL while ethanolic extracts from root were not toxic with LC_50_>100 μg/mL). Ethanol extracts from fruits and roots of *T. villosa *were found to be very toxic with LC_50 _values of 9.690 μg/mL and 4.511 μg/mL, respectively, while, ethanol extracts from leaves and twigs of *T. villosa *were found to be non toxic (LC_50_>100 μg/mL).

**Conclusion:**

These results support the use of *C. mucronata *in traditional medicine for treatment of wounds. Extracts of *C. mucronata *have potential to yield active antimicrobial and larvicidal compounds. The high brine shrimp toxicity of *T. villosa *corroborates with literature reports that the plant is toxic to both livestock and fish. The results further suggest that *T. villosa *extracts have potential to yield larvicidal and possibly cytotoxic compounds. Further studies to investigate the bioactive compounds responsible for the observed biological effects are suggested.

## Background

*Cissampelos mucronata *A. Rich (Menispermaceae) or *kishiki cha buga *(Swahili) is a dioecious liana found in tropical and subtropical areas of Africa, America and Asia [1-3]. It is widely used as traditional herbal medicine [2]. In the Coast region, Tanzania, the powdered roots of *C. mucronata *are mixed with coconut oil for treatment of fresh wounds after extraction of jigger (*Tunga penetrans*) while in other parts of Tanzania, *C. mucronata *is used for treatment of indigestion, fever due to malaria and wounds [4,5]. In South Africa, the root decoction is used for the treatment of schistosomiasis while in India roots are used as antisnake venom [6,7]. Pharmacologically root extracts of *C. mucronata *are reported to be active against chloroquine - sensitive and chloroquine - resistant *P. falciparum *strains, and active against *Trypanosoma cruzi *and *Trypanosoma rhodensiense *[5,8]. Other pharmacological properties of root extracts include sedative effect and antimicrobial activity [9,10]. Extracts from leaves are reported to have antibacterial, anti-ulcer and hypoglycaemic activities as well as uterine relaxant properties [11-13].

*Tephrosia villosa *L. Pers (Fabaceae) is an annual or perennial bushy herb which is commonly found on sandy soil. Despite being reported to be toxic to livestock and fish [14], aqueous extract of *T. villosa *leaves is used as herbal remedy in the treatment of diabetes mellitus in India [15,16]. In Africa, the herb is used as green manure to improve the soil [14].

The aim of this study was to evaluate the ethanolic and dichloromethane extracts from roots and aerial parts of *C. mucronata *as well as ethanolic extracts from leaves, fruits, twigs and roots of *T. villosa *for larvicidal and antimicrobial activity. The brine shrimp toxicity test was used to evaluate the extracts for toxicity and potential cytotoxic activity.

## Methods

### Materials

Dichloromethane was purchased from UNILAB (UNILAB^®^, Nairobi, Kenya), ethanol (absolute) was bought from Fluka Chemie GmbH (Sigma-Aldrich^®^, Zwijndrecht, Netherlands) whereas Dimethyl sulfoxide (DMSO) was purchased from Sigma^® ^(Poole, Dorset, UK). Saboraud's broth was bought from HIMEDIA^® ^(Himedia Laboratories Pvt Ltd, Mumbai, INDIA) while Nutrient broth was purchased from Tulip Diagnostic (P) Ltd (Microxpress™, Goa, INDIA). *Staphylococcus aureus *(NCTC 25923), *Escherichia coli *(ATCC 25922), *Pseudomonas aeruginosa *(ATCC 29953), *Salmonella typhi *(NCTC 8385), *Vibrio cholera *(clinical isolate), *Bacillus anthracis *(NCTC10073), *Streptococcus faecalis *(clinical isolate), *Candida albicans *(ATCC 90028) and *Cryptococcus neoformans *(clinical isolate) were obtained from the Department of Microbiolgy, Muhimbili University of Health and Allied Sciences (MUHAS). Iodonitrotetrazolium chloride was bought from SIGMA^® ^(Sigma- Aldrich^®^, St Louis, USA). The Brine Shrimps eggs were purchased from Aquaculture innovations (Grahamstown 6140, South Africa) and sea salt was prepared locally by evaporating water collected from the Indian Ocean, along the Dar es Salaam Coast. Mosquito larva, *Culex quinquefasciatus *Say., were obtained and reared at the Institute of Traditional Medicines - MUHAS.

### Collection and Preparation of Plant Materials

The roots and aerial parts (twigs and leaves) of *C. mucronata*, voucher specimen no. 3960 were collected from Bagamoyo District, while roots, twigs, leaves and fruits of *T. villosa*, voucher specimen no. 3961 was collected from Kibaha District, Coast region, Tanzania. The plants were identified by Mr. Haji Selemani, a botanist from the Department of Botany, University of Dar es Salaam. The plant materials were dried under shade and then pulverized to get powders for extraction.

### Extraction process

The plant powders from the roots and aerial parts of *C. mucronata *were extracted sequentially by maceration using dichloromethane and ethanol whereas fruits, leaves, twigs, and roots of *T. villosa *were separately extracted using ethanol only (100%). The extracts were dried under *vacuo *using rotary evaporator and stored at -20°C until the time for testing.

### Testing for larvicidal activity

The larvicidal test was performed according to World Health Organisation (WHO) protocol with minor modification [17]. Briefly, stock solutions (50 mg/mL) of each plant extract were prepared by first dissolving them in DMSO. The stock solutions were diluted with distilled water to make 100 mL each, of 500, 250, 100 and 50 μg/mL solutions of each plant extract. Ten late third instar laboratory reared *C. quinquefasciatus *mosquito larvae were then introduced in the test solution and mortality was observed after 24 h, 48 h and 72 h. Negative control tests contained mosquito larvae, DMSO (0.5%) and water only. All tests were carried out in duplicate under controlled temperature (25 ± 2°C) and relative humidity of 75-85%. The number of dead larvae was recorded after 24 h, 48 h, and 72 h, and the mean percentage mortalities calculated for each concentration. The mean results of the percentage mortality were plotted against the logarithms of concentrations using the Fig P computer program (Biosoft Inc, USA). The concentrations killing fifty percent of the larvae (LC_50_) were calculated from the regression equations obtained from the graphs.

### Testing for antimicrobial activit*y*

Minimum inhibitory concentrations (MICs) were determined by microdilution method [18] using 96-well microtitre plates. The plates were first preloaded with 50 μL of the broth media in each well followed by an addition of 50 μL of the extract (100 mg/mL) into the first wells of each row tested to make a total volume of 100 μl in the first wells. After thorough mixing 50 μl were drawn from each of the first row wells and put into the next row wells. The process was repeated down the columns to the last wells at the bottom where 50 μL was discarded. Thereafter, 50 μL of the bacterial suspension (0.5 Mac Farhland standard turbidity) was then added in each well to make the final volume of 100 μL in each well. The rows containing Gentamicin sulphate (50 - 0.024 μg/mL) was used as a standard positive drug, DMSO as negative control while the rows with broth and bacteria only was used to monitor bacterial growth. The plates were then incubated at 37°C for 24 h. For each extract, MICs were determined by adding 40 μLof 0.02% *p*-iodonitrotetrazolium (INT) chloride dye in each well followed by incubation for 1 h at 37°C. Bacterial growth was indicated by a change in pink colour. The lowest concentration which showed no bacterial growth was considered as MIC.

### Brine shrimps lethality test

Brine shrimps lethality test (BST) was used to predict the presence of bioactive compounds in the extract [19]. Briefly, stock solutions (40 mg/mL) of all extracts were prepared by dissolving them in DMSO. Different levels of concentrations (240, 120, 80, 40, 24 and 8 μg/mL) were prepared by drawing different volumes from the stock solutions and then added into vials, each containing ten brine shrimps larvae. The volume was then adjusted to 5 mL with artificial sea water prepared by dissolving 3.8 g of sea salt in 1 L of distilled water. Each level of concentration was tested in duplicate. The negative control contained brine shrimp, artificial sea water and DMSO (0.6%) only. The vials were incubated under light for 24 h. The dead larvae were counted and mean was subjected to analysis using Fig P computer program (Biosoft Inc, USA).

### Data analysis

The mean results of the percentage mortality were plotted against the logarithms of concentrations using the Fig P computer program. Regression equations obtained from the graphs were used to obtain LC_16_, LC_50_, LC_84 _and the 95% CI values [20]. An LC_50 _value greater than 100 μg/mL is considered to represent an inactive compound or extract.

## Results

### Larvicidal and Brine shrimp Activity

Larvicidal activity of ethanol and dichloromethane extracts from both roots and aerial parts of *C. mucronata *were evaluated after incubating mosquito larvae with different concentrations of extracts for 72 h. After 24 h of larvae exposure, ethanolic and dichloromethane root extracts from *C. mucronata*, CMRE and CMRD, respectively were found to have larvicidal activity whereas ethanolic and dichloromethane extracts from aerials parts of *C. mucronata*, CMAD and CMAE, respectively were found to have no activity against *C. quinquefasciatus *larvae (Table 1). However, among the active extracts, the dichloromethane root extract, CMRD exhibited high larvicidal activity with an LC_50 _value of 126.11 μg/mL than ethanolic root extracts, CMRE which had an LC_50 _value of 219.99 μg/mL. Generally the larvicidal activity increased slightly with the increase in the time of exposure except for CMAD which was found to be inactive even after 72 h of larvae exposure to different concentrations of this extract. Among the extracts from *C. mucronata*, the highest larvicidal activity was observed with CMRD extract which had an LC_50 _value of 117.09 μg/mL) after 72 h of exposure.

**Table 1:** Larvicidal activity of extracts from *C.mucronata *and *T. villosa*

Plant extracts	Larvicidal activity		
	**LC_50 _μg/ml (95% CI)**		

	**24 h (Day 1)**	**48 h (Day 2)**	**72 h (Day 3)**

CMAE	¤	26891.0(5252.15-137681.92)	7999.935 (3440.83 - 18599.8)

CMRE	219.99 (180.91 - 267.51)	217.12 (205.77 - 229.60)	207.12 (196.00 -219.01)

CMAD	¤	¤	¤

CMRD	126.11 (104.14 - 152.72)	122.91 (101.33 - 149.09)	117.09 (95.59 - 143.44)

TVFE	65.75 (51.58 - 77.78)	54.465 (48.022 - 59.713)	53.25 (45.67 - 62.09)

TVLE	213.59 (201.05 - 227.59)	169.75 (155.34 - 185.03)	161.61 (147.99 - 176.07)

TVTE	126.16 (105.70 - 145.21)	74.724 (66.798 - 82.347)	71.171 (63.739 - 78.072)

TVRE	218.47 (198.88 - 238.97)	107.61 (89.26 - 124.36)	71.50 (54.11 - 86.15)

**¤ **= No mortality at all levels of concentration tested, CI = confidence interval, CMAE = *Cissampelos mucronata *aerial parts extract (100% ethanol), CMRE = *Cissampelos mucronata *root extract (100% ethanol), CMAD = *Cissampelos mucronata *aerial parts extract (dichloromethane), CMRD = *Cissampelos mucronata *root extract (dichloromethane), TVFE = *Tephrosia villosa *fruits extract (100% ethanol), TVLE = *Tephrosia villosa *leaves extract (100% ethanol), TVTE = *Tephrosia villosa *twigs extract (100% ethanol), TVRE = *Tephrosia villosa *root extract (100% ethanol).

All extracts from *T. villosa *showed larvicidal activity (Table 1) against mosquito larvae after 24 h of incubation with LC_50 _values ranging from 65 μg/mL to 220 μg/mL. However, after this time of exposure, ethanolic fruit extract from *T. villosa*, TVFE, exhibited the best larvicidal activity with LC_50 _value of 65.75 μg/mL as compared to other extracts from *T. villosa*. Larvicidal activity was increased with the time of exposure. At day three, TVFE was found to be more active against *C. quinquefasciatus *larvae with an LC_50 _value of 53.25 μg/mL while the ethanolic leaf extract from *T. villosa *(TVLE), was found to be less active with an LC_50 _value of 161.61 μg/mL.

Brine shrimps results as indicated in Table 2 show that all extracts from aerial parts and ethanolic root extract from *C. mucronata *were not toxic to brine shrimp larvae, giving LC_50 _values above 100 μg/mL. The root dichloromethane extracts of *C.mucronata *was found to be the most toxic to brine shrimp (LC_50 _= 59.608 μg/mL). Ethanolic extracts of the fruits (TVFE) and roots (TVRE) of *T.villosa*, were highly toxic to brine shrimp larvae with LC_50 _values below 10 μg/mL. The ethanolic root extract of *T. villosa *was however the most toxic extract with an LC_50 _value of 4.5 μg/mL. Ethanolic extracts from leaves (TVLE) and twigs (TVTE) were non toxic with LC_50 _values above 100 μg/mL (Table 2).

**Table 2:** Brine shrimp activity of extracts from *C.mucronata *and *T. villosa*

Plant extracts	Regression equation	LC_50 _(μg/ml)	95% Confidence interval (CI)	Regression coefficient (r)
CMAE	Y = 65.1169logx - 86.7002	125.691	90.101 6 - 175.339	0.926045

CMRE	Y = 114.1741logx - 196.0862	143.008	115.608 - 176.901	0.918404

CMAD	Y = 90.1643logx - 135.5126	114.155	113.329 - 145.205	0.935504

CMRD	Y = 85.4278logx - 101.6604	59.608	46.244 - 76.835	0.975568

TVFE	Y = 68.4308logx - 17.4949	9.690	7.058 - 13.304	0.968423

TVLE	Y = 127.7823logx - 221.9319	134.304	111.087 - 162.373	0.899348

TVTE	Y = 68.0408logx - 111.7285	238.196	166.687 - 340.382	0.969139

TVRE	Y = 95.0056logx - 12.1626	4.511	3.589 - 5.670	0.999793

Cyclophosphamide	Y = 69.9680logx - 34.9360	16.365	12.006 - 22.305	0.994929

CMAE = *Cissampelos mucronata *aerial parts extract (100% ethanol), CMRE = *Cissampelos mucronata *root extract (100% ethanol), CMAD = *Cissampelos mucronata *aerial parts extract (dichloromethane), CMRD = *Cissampelos mucronata *root extract (dichloromethane), TVFE = *Tephrosia villosa *fruits extract (100% ethanol), TVLE = *Tephrosia villosa *leaves extract (100% ethanol), TVTE = *Tephrosia villosa *twigs extract (100% ethanol), TVRE = *Tephrosia villosa *root extract (100% ethanol).

### Antimicrobial Activity

All the extracts from *C. mucronata *and *T. villosa *exhibited activity against at least one organism tested (Table 3). *C. mucronata *aerial parts and root extracts were found to be active against both bacteria and fungi. The aerial parts dichloromethane and ethanolic extracts exhibited the highest activity. The ethanolic aerial part extracts of *C. mucronata *was most active against *V. cholera *with MIC value of 0.391 mg/mL while the dichloromethane of the same was more active against *S. typhi, B. anthracis and C. neoformans with *MIC values of 0.781 mg/mL, 0.195 mg/mL and 0.195 mg/mL, respectively. The ethanolic root extract from *C. mucronata *exhibited low activity against *B. anthracis*, *V. cholera*, *S. faecalis*, *C. albicans *and *C. neoformans *with MIC values ranging between 1.563 mg/mL and 3.125 mg/mL (Table 3).

**Table 3:** Antimicrobial activity of extracts from *C.mucronata *and *T. villosa*

Plant extracts	minimum inhibitory concentration, MIC (mg/mL)								
	***S. aureus***	***E. coli***	***P. aeruginosa***	***S. typhi ***	***V. cholera***	***B. anthracis***	***S. faecalis***	***C. albicans***	***C. neoformans***

CMAE	3.125	6.250	6.250	3.125	0.391	3.125	12.5	1.563	1.563

CMRE	12.50	6.250	12.50	6.250	3.125	3.125	3.125	3.125	1.563

CMAD	6.250	6.250	12.50	0.781	3.125	0.195	6.25	3.125	0.195

CMRD	> 25.0	> 25.0	> 25.0	> 25.0	> 25.0	> 25.0	> 25.0	> 25.0	> 25.0

TVFE	6.25	12.50	12.5	3.125	3.125	1.56	12.5	12.5	0.195

TVLE	12.50	0.781	1.563	3.125	12.50	1.563	12.50	1.563	6.25

TVTE	6.250	12.50	12.50	3.125	6.250	12.50	12.50	12.5	1.563

TVRE	1.563	12.50	12.50	3.125	6.250	0.195	3.125	12.50	6.25

Standard antibacterial drug (Gentamicin)	0.000781	0.003125	0.001563	0.00625	0.001563	0.003125	0.0000244	NA	NA

CMAE = *Cissampelos mucronata *aerial parts extract (100% ethanol), CMRE = *Cissampelos mucronata *root extract (100% ethanol), CMAD = *Cissampelos mucronata *aerial parts extract (dichloromethane), CMRD = *Cissampelos mucronata *root extract (dichloromethane), TVFE = *Tephrosia villosa *fruits extract (100% ethanol), TVLE = *Tephrosia villosa *leaves extract (100% ethanol), TVTE = *Tephrosia villosa *twigs extract (100% ethanol), TVRE = *Tephrosia villosa *root extract (100% ethanol), NA = applicable.

Among the *T.villosa extracts t*he best activity was exhibited by the fruit extracts against *C.neoformans *with MIC value of 0.195 mg/mL, leaf extract against *E. coli *with MIC value of 0.781 mg/mL and roots extract against *B.anthracis *with MIC value of 0.195 mg/mL. Most of the organisms tested were less sensitive to the ethanolic twig extract (TVTE) except *C. neoformans *(MIC = 1.563 mg/mL) and *S. typhi *(MIC = 3.125 mg/mL).

## Discussion

Leaves and roots of *C. mucronata *are used by some communities to treat venereal diseases, wounds and gastro-intestinal complaints such as diarrhoea and dysentery [1,8]. *T. villosa *is reported to be toxic to animals and fish; however, in India it is used in the treatment diabetes mellitus [16]. There is no larvicidal activity is reported before for *C. mucronata *and *T. villosa*. Therefore, in this study, larvicidal, antimicrobial and brine shrimp activities were evaluated to determine the medicinal value and safety of these plant species.

The larvicidal activity assay was used to predict the presence of bioactive compounds that are able to kill mosquito larvae (*C. quinquefasciatus *larvae) or disrupt their development towards the adult form which is responsible for disease transmission [17]. Similarly, the brine shrimp lethality test is normally used to predict presence of toxic bioactive compounds but also possible presence of compounds with potential anticancer activity [21]. In this regard, ethanol and dichloromethane root extracts from *C. mucronata *exhibited high larvicidal activity, with the highest activity observed with dichloromethane extract which had the LC_50 _value of 117.09 μg/mL. *C. mucronata *aerial part extracts did not exhibit any larvicidal activity. The extract is generally regarded as non-toxic if its LC_50 _is greater than 100 μg/mL in the brine shrimp lethality assay [21]. In this case, ethanolic extract of the roots of *C. mucronata *had larvicidal activity but not toxic, while dichloromethane extract showed larvicidal activity but also observed to be toxic. The literature report that the root extract of *C. mucronata *is active against chloroquine-sensitive and chloroquine-resistant *P. falciparum *malaria parasites [5], which is transmitted by Anopheles mosquito. Mosquitoes are responsible for the spread of various vector born diseases including malaria which is found in more than 100 countries in the world [22]. Although there are several ways of preventing the mosquito borne-diseases, one of the best option to prevent the spread of those diseases is to control mosquito vector by using larvicides. However, resistance and environment damage caused by synthetic agents have prompted for the search for environmental friendly and effective larvicides from plant sources [23]. In this study, results have shown that ethanol extract of the roots of *C. mucronata *may be useful as larvicidal agent.

Based on arguments by Aligiannis et al., 2001 [24], extracts from *C. mucronata *with promising antimicrobial activity were CMAE against *V. cholera *(MIC = 0.391 mg/mL) and CMAD against *B. anthracis *(MIC = 0.195 mg/mL), *S.typhi *(MIC = 0.781 mg/mL), and *C. neoformans *(MIC = 0.195 mg/mL). Antimicrobial activities of *C. mucronata *collected from other parts of the world have already been documented [10,11]. Although *C. mucronata *roots are used by the people of Coast region in Tanzania for treatment of fresh wounds, the antimicrobial activity of the extracts from roots was found to be generally lower than that of the extracts from aerial parts (Table 2). Therefore, the use of root extracts for treatment of fresh wound may suggests that, apart from the weak antibacterial properties, *C. mucronata *root extract might has other properties such as anti-inflammatory property. Furthermore, these results suggest that, the aerial parts of this plant species may substitute roots in the preparation of herbs needed in the treatment of wounds and other bacterial infection.

All ethanolic extracts *of T. villosa *exhibited larvicidal activity, with highest activity being in the fruit extract which had LC_50 _value of 53.25 μg/mL. Furthermore, extracts from leaves and twigs were not toxic to brine shrimp (LC_50 _> 100 μg/mL) whereas extracts from fruits and roots were very toxic to brine shrimp with LC_50 _< 10 μg/mL (Table 1). Interestingly, the LC_50 _value of twigs extract in the larvicidal activity was lower than the LC_50 _for brine shrimp toxicity. This suggests that, ethanolic extract from twigs may be useful as larvicides in the control of the spread of mosquito-borne diseases. This observation corroborates the earlier reports on the seeds of *T. vogelii *which share the same genus as *T. villosa*. The seeds of *T. vogelii *are reported to have larvicidal activity against *Aedes aegti *larvae [25].

Brine shrimp results obtained from fruits and roots support previous claims of *T. villosa *toxicity to animals and fish [14] and medicinal use of leaf extract (16). Fruit and root extracts of *T. villosa *were found to be very toxic with their LC_50 _values below that of the anticancer drug, cyclophosphamide whereas leaf and twig extracts were found to be non-toxic (Table 1). This suggests that the toxic characteristic of *T. villosa *is due to the fruits and roots, and strengthen the speculation for potential to yield anticancer agents (26).

The ethanolic extracts from *T. villosa *were also evaluated for their antimicrobial properties. The extracts with promising antimicrobial activity were from fruits against *C.neoformans *with MIC = 0.195 mg/mL, roots against *B. anthracis *with MIC = 0.195 mg/mL and leaves against *E. coli *with MIC = 0.781 mg/mL. When compared with other species in the same genus, most of the species reported earlier showed no or weak antimicrobial properties [26]. Some of the reported antimicrobial activities from the genus *Tephrosia *are of methanolic extract from aerial parts of *T. apollinea *which was active against *C. albicans *and of aqueous extract from the entire *T. purpurea *which was active against *S. aureus *[27,28]. Although most of the extracts from *T. villosa *showed weak antibacterial and antifungal activity, the good activity observed from the fruit extract against *C. neoformans *suggests that further studies is needed to identify bioactive compound responsible for the antimicrobial activity.

## Conclusion

These results support the use of *C. mucronata *in traditional medicine for treatment of wounds. Extracts of *C. mucronata *have potential to yield active antimicrobial and larvicidal compounds. The high brine shrimp toxicity of *T. villosa *corroborates with literature reports that the plant is toxic to both livestock and fish. The results further suggest that *T. villosa *extracts have potential to yield larvicidal and possibly cytotoxic compounds. Further studies to investigate the bioactive compounds responsible for the observed biological effects are suggested.

## Competing interests

The authors declare that they have no competing interests.

## Authors' contributions

RSON: was responsible for study concept, study design, methodological development, data acquisition and data analysis, and writing the manuscript. ZHM: was responsible for coordinating the research activities and writing the manuscript. AWK: was responsible for study concept, study design, methodological development and data acquisition. EMI: was responsible for methodological development, coordinating the research and revising the manuscript. MJM: was involved in coordinating the study and revising the manuscript. PE: was involved in methodological development and revising the manuscript. MJM: was involved in coordinating the study and revising the manuscript. All authors read and approved the final manuscript.

## Pre-publication history

The pre-publication history for this paper can be accessed here:

http://www.biomedcentral.com/1472-6882/11/33/prepub
